# SP-8356, a (1S)-(–)-verbenone derivative, exerts *in vitro* and *in vivo* anti-breast cancer effects by inhibiting NF-κB signaling

**DOI:** 10.1038/s41598-019-41224-y

**Published:** 2019-04-29

**Authors:** Sunam Mander, Dong Hwi Kim, Huong Thi Nguyen, Hyo Jeong Yong, Kisoo Pahk, Eun-Yeong Kim, Kiho Lee, Jae Young Seong, Won-Ki Kim, Jong-Ik Hwang

**Affiliations:** 1 Departments of Biomedical Sciences, College of Medicine, Korea University, 73 Inchon-ro, Seongbuk-gu, Seoul, 136-705 Republic of Korea; 2 Departments of and Neuroscience, College of Medicine, Korea University, 73 Inchon-ro, Seongbuk-gu, Seoul, 136-705 Republic of Korea; 3 Institute of Inflammation Control, Korea University, 73 Inchon-ro, Seongbuk-gu, Seoul, 02841 Republic of Korea; 4 Department of Nuclear Medicine, Korea University, Anam Hospital, 73 Inchon-ro, Seongbuk-gu, Seoul, 02841 Republic of Korea; 5 College of Pharmacy, Korea University, Sejong, 30019 Republic of Korea; 6 Biomedical Research Center, Korea University Guro Hospital, Seoul, 08308 Republic of Korea

**Keywords:** Breast cancer, Target identification

## Abstract

Breast cancer exhibits high lethality in women because it is frequently detected at an advanced stage and aggressive forms such as triple-negative breast cancer (TNBC), which are often characterized by metastasis through colonization of secondary tumors. Thus, developing therapeutic agents that target the metastatic process is crucial to successfully treat aggressive breast cancer. We evaluated SP-8356, an anti-inflammatory synthetic verbenone derivative, with respect to its regulation of breast cancer cell behavior and cancer progression. Treatment of SP-8356 arrested cell cycle and reduced growth in various types of breast cancer cells with mild cytotoxicity. Particularly, SP-8356 significantly reduced the motility and invasiveness of TNBC cells. Assays using an *in vivo* xenograft mouse model confirmed the cell-specific anti-proliferative and anti-metastatic activity of SP-8356. Functional studies revealed that SP-8356 suppressed serum response element-dependent reporter gene expression and NF-κB-related signaling, resulting in downregulation of many genes related to cancer invasion. We conclude that SP-8356 suppresses breast cancer progression through multimodal functions, including inhibition of NF-κB signaling and growth-related signaling pathways.

## Introduction

Breast cancer is one of the most fatal diseases in women and exhibits high incidence and mortality among various cancers^1,2^. In addition, global burden of breast cancer surpasses that of other cancers and incidence rate is increasing. Although early detection and appropriate treatment with surgical resection increase survival rate, detection in advanced stages and diagnosis of atypical triple-negative breast cancer (TNBC) usually result in poor therapeutic outcomes with high lethality. The most common causes of death related breast cancer are metastasis and subsequent colonization of secondary tumors in other organs or tissues. Extensive studies of cellular signaling networks have identified proteins necessary for metastasis and uncontrolled growth of cancers, which has led to the development of targeted therapies against cancer-driving molecules in the last decade^3^. However, because of its heterogeneous nature, inherent chromosomal instability, and the complex context of tumor environments, breast cancers may easily acquire resistance to targeted drugs. Thus, to overcome the limitations of current therapies, new effective drug identification and development are necessary^4–7^.

Among various signaling pathways which promote tumor growth and progression, an inflammatory transcription factor NF-κB plays pivotal roles on cell proliferation and survival. Interestingly, NF-κB has been reported to be highly activated in various types of cancers, including breast, bladder, prostate carcinoma and melanoma^8–10^. In breast cancer, aberrant constitutively active NF-κB exacerbates malignancy without hormonal dependency^11^. IKKβ is an essential kinase in the canonical NF-κB activation pathway that induces phosphorylation and degradation of IκB and subsequent nuclear translocation of NF-κB. According to previous reports, IKKβ is overexpressed in some types of cancers, potentially implicating NF-κB signaling in cancer progression^12,13^. Thus, suppression of NF-κB and components of its canonical activation pathway may provide useful targets for anti-cancer agents.

Previously, essential oils containing (1S)-(–)-verbenone was reported to have anti-inflammatory effects^14^. Thus, we have synthesized a series of (1S)-(–)-verbenone derivatives to enhance their cytoprotective effects with stronger anti-inflammatory and anti-oxidant activities^15^ and found that certain (1S)-(–)-verbenone derivatives inhibited cell motility via inhibition of NF-κB signaling pathways (unpublished results). One of these (1S)-(–)-verbenone derivatives, (1S,5 R)-4-(3,4-dihydroxy-5-methoxystyryl)-6,6-dimethylbicyclo[3.1.1]hept-3-en-2-one (SP-8356; compound 3 f reported in our previous paper^15^), was found most effective in blocking the cell motility, which is recognized as a critical parameter of anti-metastatic activity in cancer research.

In the present study, therefore, we investigated the effects of SP-8356 on breast cancer cells and demonstrated that SP-8356 inhibits their growth by regulating cell cycle progression and limiting their motilities. Our functional studies also indicated that SP-8356 suppress breast cancer metastasis by targeting various cellular targets, such as NF-κB signaling.

## Results

### SP-8356 inhibits breast cancer cell proliferation by mild cytotoxicity and cell cycle arrest

**Figure 1:**
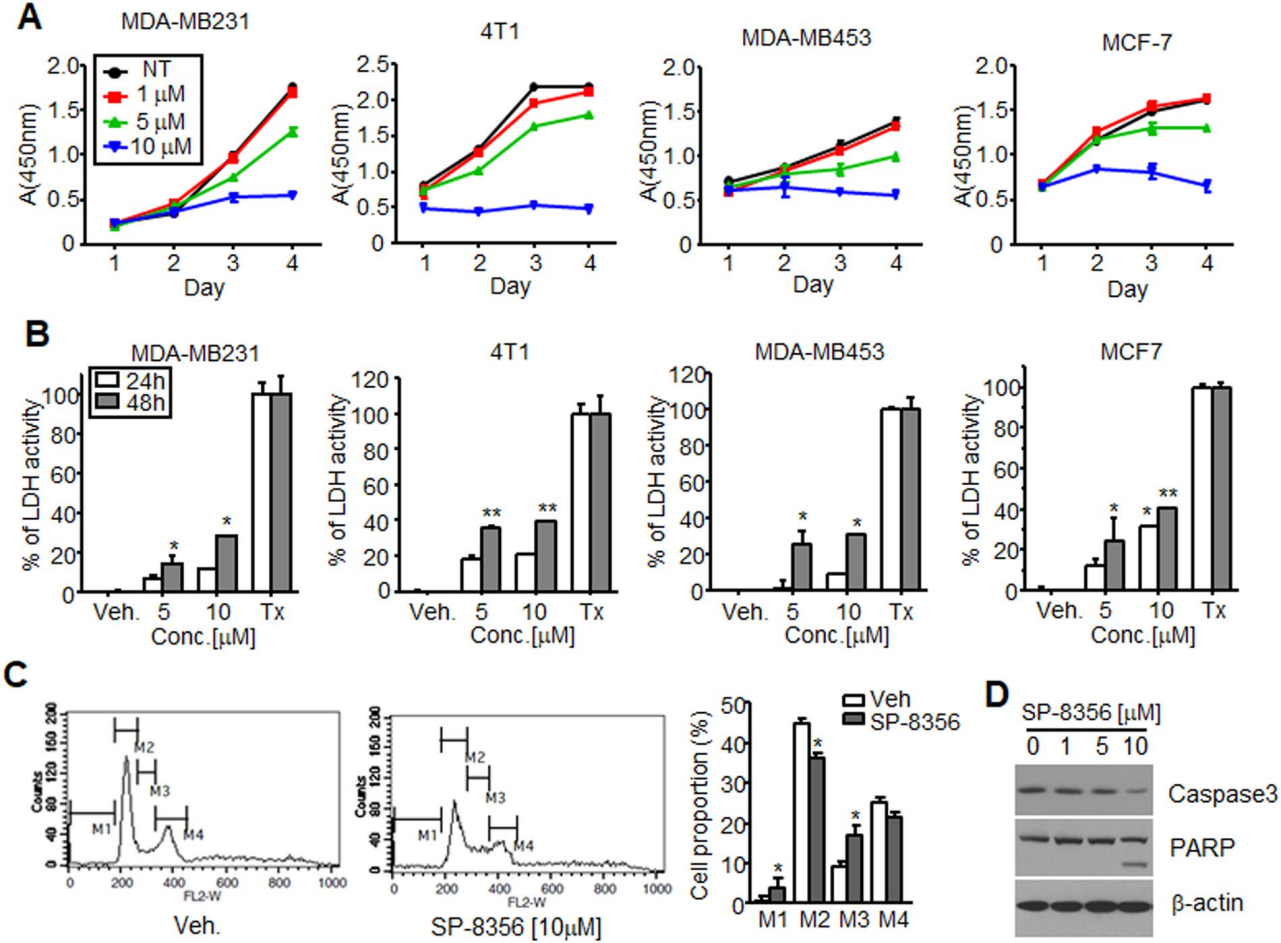
SP-8356 inhibits growth of breast cancer cells. (**A**) Breast cancer cells were exposed to varying concentrations of SP-8356 and analyzed by CCK-8 assays. Values are shown as means ± SEM. (**B**) Cell death in MDA-MB231 cells mediated by SP-8356 was assessed based on LDH levels. As a positive control, cells were lysed with 1% Triton X-100. Values are shown as means ± SEM. *p < 0.05, **p < 0.01 (against vehicle treatment) (**C**) Cell cycle progression of MDA-MB231 cells after exposure to 10 μM SP-8356 or vehicle (DMSO) for 48 h based on propidium iodide staining of DNA contents. M1, apoptotic cells; M2, G_0_/G_1_ phase; M3, S phase; M4, G_2_/M phase. Values are shown as means ± SEM. *p < 0.05 (against vehicle treatment) (**D**) Effect of SP-8356 on activity of apoptotic markers. Cells were treated with different doses of SP-8356 for 48 h, and 20 μg of clarified lysates were applied for separated SDS-PAGE gels and analyzed by Western blotting with appropriate antibodies (β-actin served as the positive control). Exposure time during development with ECL solution; 30 sec. for Caspase3 and PARP, 15 sec. for β-actin).

To explore the anti-cancer activities of (1S)-(–)-verbenone derivatives, we screened 10 synthesized molecules for growth inhibition of MDA-MB231 breast cancer cells and found that SP-8356 significantly limited their proliferation (data not shown). We further determined that SP-8356 inhibited growth of various types of breast cancer cell lines, including MDA-MB231, MDA-MB453, 4T1, and MCF-7 cells, in a time- and dose-dependent manner (Fig. 1A). To investigate if the reagent induced cytotoxicity, cell death was analyzed in LDH assays. Treatment with 5 and 10 μM SP-8356 for 48 h led to 14.48% and 28.7% cytotoxicity, respectively, in MDA-MB231 cells, 35.31% and 29.65% in 4T1 cells, 25.53% and 31.07% in MDA-MB453 cells, and 24.73% and 30.93% MCF-7 cells (Fig. 1B). Cell cycle analysis revealed an increase of SP-8356-treated MDA-MB231 cells in sub-G_0_ phase compared to the control group, denoting possible apoptosis in the experimental group. We also observed an increased percentage of MDA-MB231 cells in S phase after SP-8356 treatment at 10 μM (Fig. 1C). Because SP-8356 exhibited mild cytotoxicity, apoptosis occurrence was also evaluated by caspase-3 and PARP cleavage detection through Western blotting (Fig. 1D). Obvious decrease of casapas-3 and cleaved PARP were observed at 10 μM SP-8356 for 48 h treatment to MDA-MB231 cells, which may be inferred slightly higher toxicity at this concentration in LDH assay. Taken together, these results indicate that SP-8356 likely induces cell cycle arrest at S phase and triggers apoptosis in a percentage of treated cells.

### SP-8356 downregulates migration and invasion of TNBC cells

**Figure 2:**
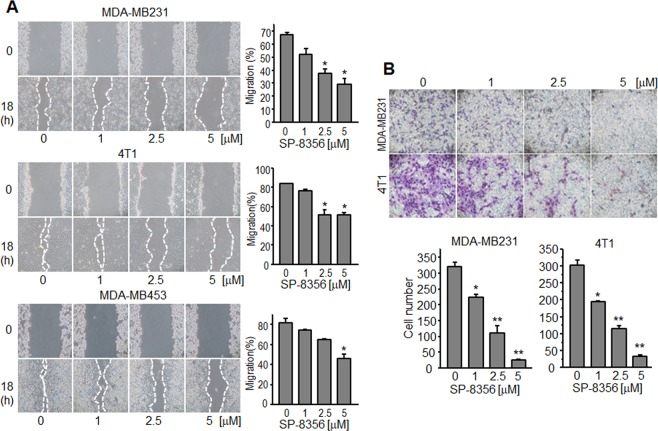
SP-8356 inhibits migration and invasion of breast cancer cells. (**A**) Wound healing assays. MDA-MB231, 4T1, and MDA-MB453 cells were cultured to near confluence and incubated with indicated doses of SP-8356 after wounding with a pipette tip. Images were captured at 0 h and 18 h. Right panels show percentages of wound closure in the various treatment groups. Values are shown as means ± SEM. (**B**) Invasion assays. MDA-MB231 and 4T1 cells treated with SP-8356 were loaded into Matrigel-coated transwell chambers and incubated for 24 h. Cells that migrated to the lower membrane surface were fixed and stained. Representative images of treatment with each concentration of SP-8356 are shown. Invading cells were counted in five high-power microscope fields and averaged. Values are shown as means ± SEM. *p < 0.05, **p < 0.001.

Because most TNBCs are highly aggressive and account for the major proportion of breast cancer deaths due to metastasis^16,17^, we examined the effect of SP-8356 on cell migration by performing a wound healing assay with varying doses of the molecule. Wound gaps in the 2.5 μM and 5 μM SP-8356-treated groups were significantly wider than those of the untreated groups in MDA-MB231, 4T1, and MDA-MB453 cells (Fig. 2A). We also assessed the molecule’s effect on cell motility and found that SP-8356 significantly reduced invasion of MDA-MB231 and 4T1 cells in a dose-dependent manner (Fig. 2B).

### SP-8356 inhibits tumor growth and lung metastasis in a xenograft mouse model

**Figure 3:**
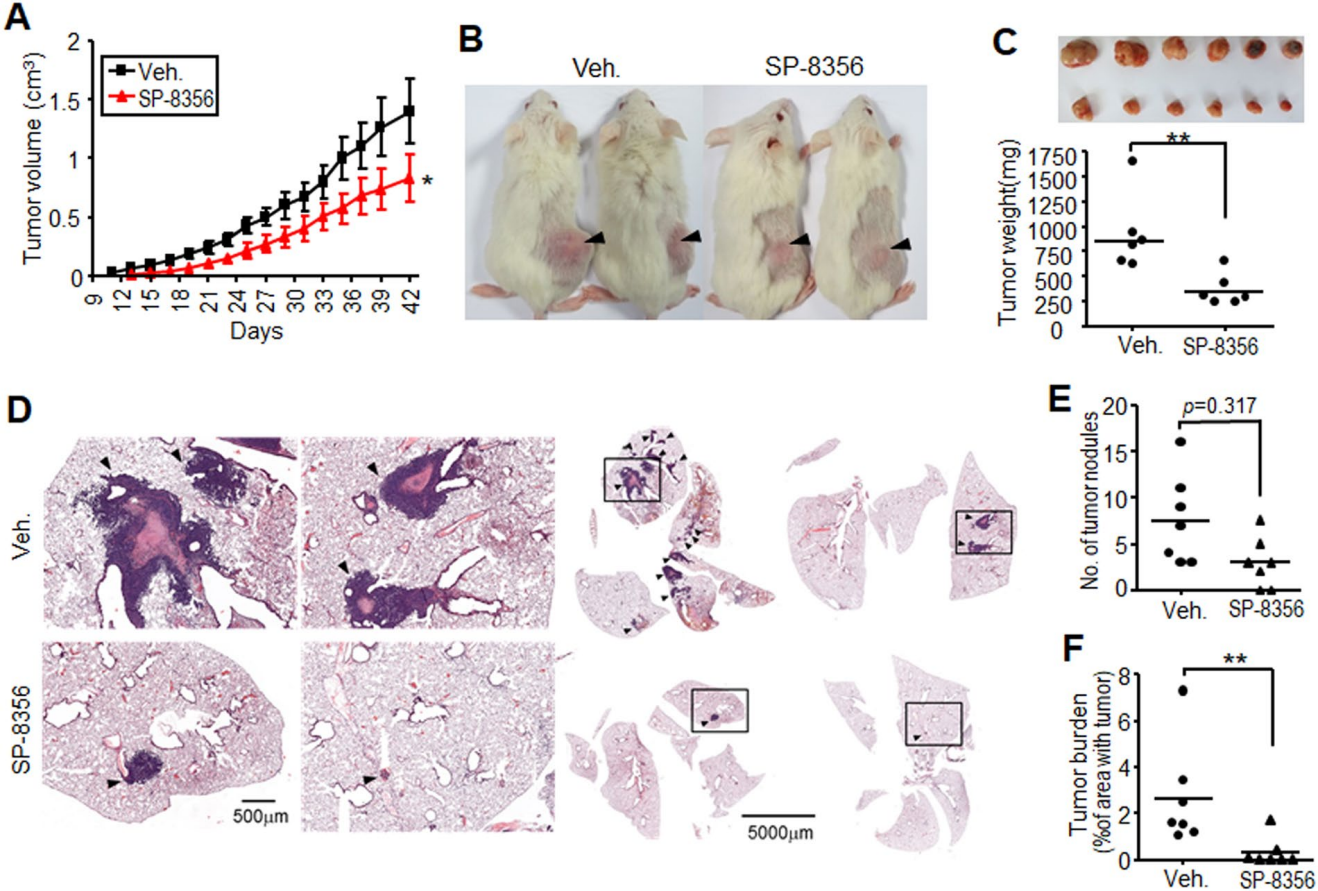
SP-8356 suppresses *in vivo* tumor growth and metastasis of MDA-MB231 breast cancer cells. (**A**) Tumor volumes of MDA-MB231 xenografts in NOD/SCID mice. Mice were treated every day with SP-8356 or vehicle, and tumors were measured every three days until the 42^nd^ day. Values are shown as means ± SEM; n = 7 mice per group, *p < 0.05. (**B**) Images of vehicle- or SP-8356-treated NOD/SCID mice with visible tumors (marked with black arrow) at the end of the treatment schedule. (**C**) Tumor weight analysis of vehicle- or SP-8356-treated mice (n = 6). (**D**) Representative images of lung sections from mice injected daily with vehicle or SP-8356 for 45 days. Arrowheads indicate tumor nodules. Scale bar, 500 μm. (**E**,**F**) Quantitation of tumor nodules and tumor burden per lung section. Values are shown as means ± SEM; n = 7 mice per group. **p < 0.001.

To investigate the *in vivo* relevance of our cellular findings, we generated tumor-induced mice via MDA-MB231 cell implantation that were injected with SP-8356 or a vehicle control. The tumor volumes of mice treated with SP-8356 were significantly lower than those of vehicle-treated mice after 42 days (Fig. 3A,B). Figure 3C shows substantially lower tumor weights in the SP-8356-treated mice than in the vehicle group, confirming SP-8356 inhibition of breast cancer cells also occurs *in vivo*. The ability of SP-8356 to potently block *in vitro* invasion of the breast cancer cells led us to investigate its effectiveness in limiting *in vivo* metastasis. Since metastatic model using orthotopic graft to mammary fat pad is not applicable for MDA-MB231 cells, cells were injected to tail vein, which is currently acceptable lung metastasis model. Lungs isolated from the xenograft mice treated with SP-8356 exhibited significantly reduced tumor burdens compared to the vehicle-treated group (Fig. 3D,E). The numbers of tumor nodules were also decreased in SP-8356-treated mice (Fig. 3F). To investigate if either the vehicle or SP-8356 itself adversely affected the mice, we applied the reagents to naïve mice for the same time period. Blood and gross anatomical analysis revealed no apparent abnormalities (data not shown), implying that SP-8356 is potentially safe in mice. Taken together, our results suggest that SP-8356 downregulates metastasis and progression of breast cancer in a cell- and tissue-specific manner.

### SP-8356 inhibits transcription factors related to growth and motility of breast cancer cells

**Figure 4:**
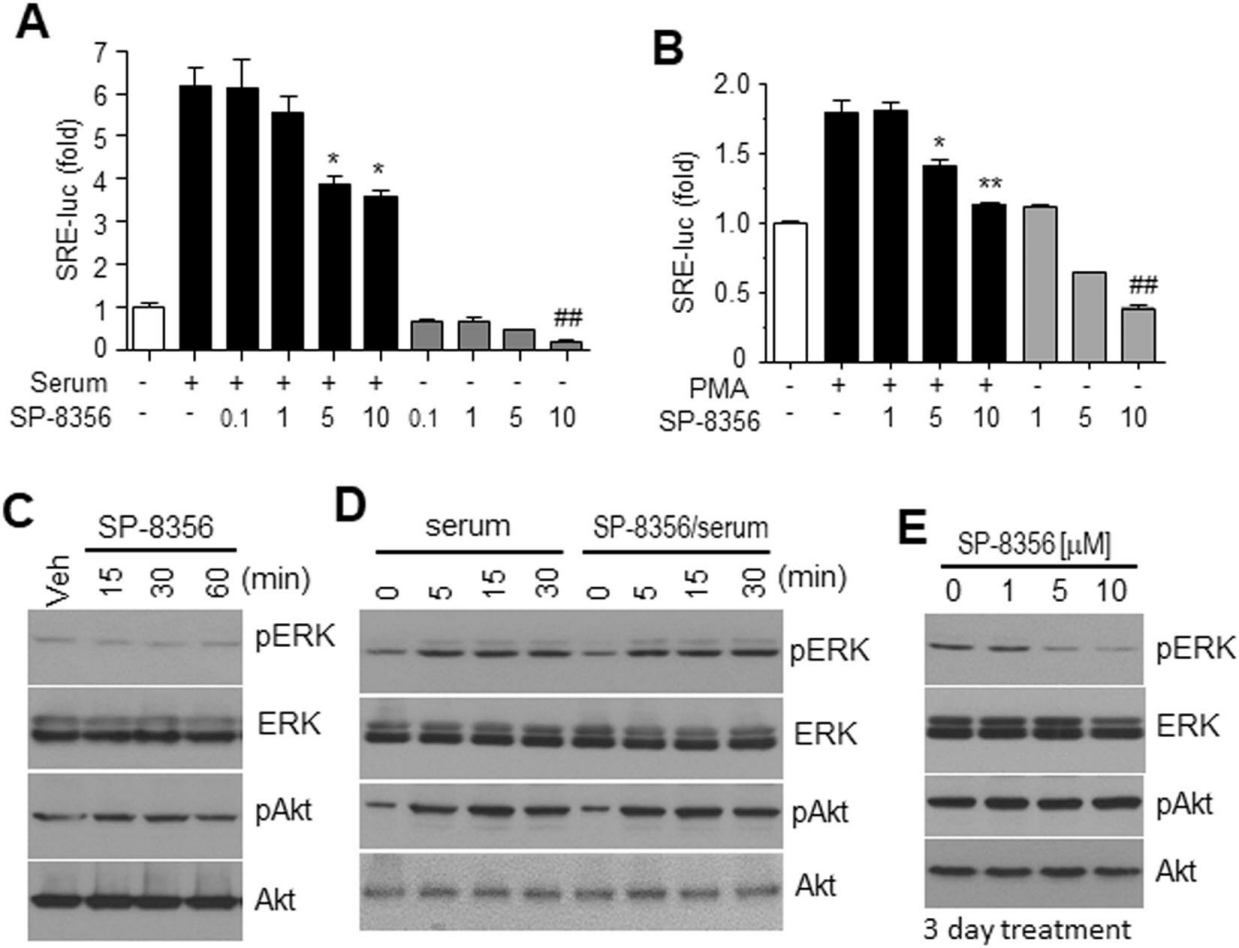
SP-8356 decreases basal ERK activity. (**A**,**B**) SP-8356 suppresses serum- or PMA-stimulated SRE activity. MDA-MB231 cells were transfected with plasmids containing a *SRE-luciferase* reporter gene construct. After 24 h of serum starvation, cells were treated with different doses of SP-8356 prior to stimulation with 10% FBS or 1 μM PMA, lysed, and analyzed in luciferase activity assays. Values are shown as means ± SEM. *p < 0.05, *p < 0.001 (compared to serum stimulation without SP-8356), ^##^*p* < 0.001 (compared to no serum or SP-8356 treatment) (**C**) To examine the effect of short time treatment with SP-3856, after 24 h starvation, MDA-MB231 cells were exposed to 10 μM SP-8356 for the indicated time points, and phosphorylation of ERK and Akt was determined by Western blotting. Veh: vehicle treatment (**D**) After 24 h serum starvation, cells were treated with 10 μM SP-8356 and stimulated with serum for the indicated times, after which lysates were subjected to Western blotting. (**E**) MDA-MB231 cells were treated with SP-8356 under normal culture conditions (no serum stimulation) for three days, and lysates were analyzed with Western blotting. (**C**,**D**,**E**) 20 μg of cell lysates was used for SDS-PAGE. After transfer, nitrocellulose membranes were divided to two parts for Western blotting against pERK and pAkt around 55 kDa region. After first blotting, antibodies were removed from the nitrocellulose membrane by detaching solution, then the membranes were used for re-blotting for ERK or Akt. Exposure time for each blotting was around 15 sec.

Aberrant activation of PI3K/AKT and Ras/MAPK pathways are associated with initiation and progression of breast cancer^18,19^; therefore, we explored the effect of SP-8356 on these pathways. Reporter gene assays revealed that SP-8356 slightly decreased activity of serum-stimulated SRE (Fig. 4A) and PMA-regulated SRE (Fig. 4B) in MDA-MB231 cells. In the absence of serum, SP-8356 downregulated basal expression of the reporter gene, although SP-8356 itself did not change basal or serum-dependent phosphorylation of ERK and Akt (Fig. 4C,D). Interestingly, in MDA-MB231 cells treated with SP-8356 under normal culture conditions, phospho-ERK levels decreased in a dose-dependent manner without any change in Akt phosphorylation (Fig. 4E), implying that SP-8356-induced growth inhibition may be partly associated with its interaction with ERK pathways.

**Figure 5:**
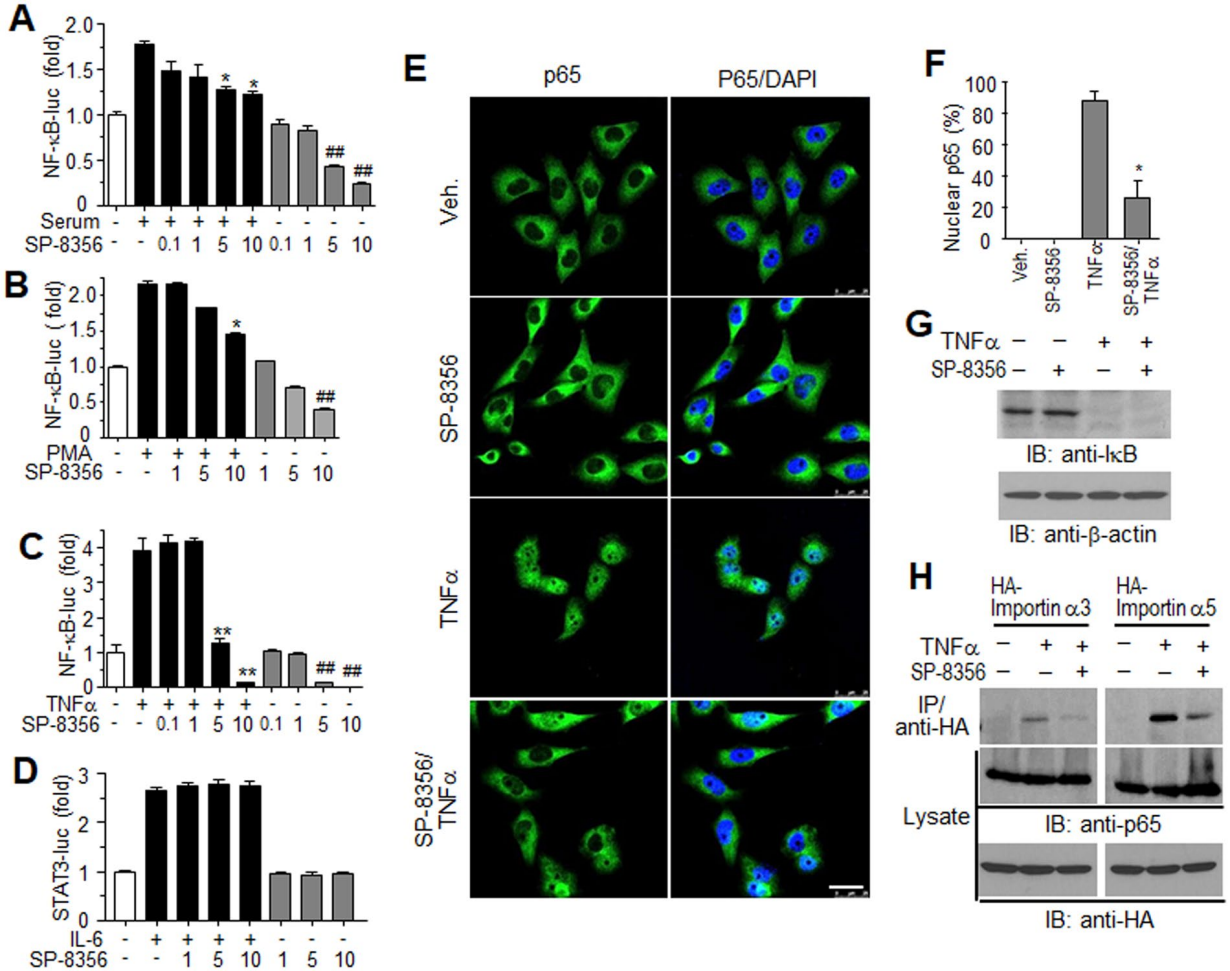
SP-8356 inhibits NF-κB-mediated signaling in MDA-MB231 cells. Cells were transiently transfected with a *NF-*κ*B-luciferase* or *STAT3-luciferase* reporter gene construct. After 24 h of serum starvation, cells were pre-treated for 30 min with SP-8356 and stimulated with 10% fetal bovine serum (**A**), 1 μM PMA (**B**), 10 ng/ml TNF-α (**C**), or 10 ng/ml IL-6 (**D**) for 6 h. Cell lysates were then assayed for luciferase activity. Values are shown as means ± SEM. *p < 0.05, **p < 0.001 (compared to agonist stimulation without SP-8356), ^##^*p* < 0.001 (compared to no stimulation or treatment) (**E**) Effect of SP-8356 on nuclear localization of the NF-κB p65 subunit. MDA-MB231 cells were pre-treated with 10 μM SP-8356 and stimulated by 10 ng/ml TNFα, after which cell nuclei (blue) and subcellular localization of p65 (green) were visualized with respective DAPI or FITC-conjugated antibodies (**F**) Quantitation of NF-κB p65 nuclear translocation in the indicated treatment groups. Results are expressed as percentages of cells in which p65 translocated to the nucleus vs. the total number of cells. Values are shown as means ± SEM. *p < 0.05. (**G**) SP-8356 has no effect on TNF-α-stimulated IκB degradation. MDA-MB231 cells were incubated with SP-8356 prior to TNF-α treatment. 20 μg of cell lysates was then subjected to Western blotting using anti-IκB antibodies (exposure time: 2 min). (**H**) SP-8356 inhibits the interaction between p65 and importin α proteins. MDA-MB231 cells expressing HA-importin α3 or α5 were treated with SP-8356 and TNF-α. Cell lysates were then subjected to immunoprecipitation with anti-HA agarose and Western blotting with antibodies against HA-epitope complexes or p65. 5% of total cell lysates was used for direct loading. Exposure time: 5 min for p65(blot from same gel was cut and relocated), 1 min for HA-tagged proteins).

NF-κB signaling is necessary for cell survival and apoptosis and has been implicated in cancer pathogenesis^8,20^. Because NF-κB activation is regulated by SP-8356 under certain conditions, including inflammation^21^, we investigated its effect on NF-κB in MDA-MB231 cells. Serum stimulation increased reporter gene activity 2-fold, while SP-8356 decreased NF-κB activity in a dose-dependent manner (Fig. 5A). We then treated the cells with PMA (protein kinase C activator), as some protein kinase C subgroups can activate IKK, an upstream kinase of NF-κB activation. As shown in Fig. 5B, SP-8356 downregulated PMA-dependent NF-κB activation. SP-8356 also significantly decreased TNFα-stimulated NF-κB activity (Fig. 5C). Interestingly, all related experiments revealed that NF-κB activity in unstimulated cells was significantly decreased in the presence of more than 5 μM SP-8356. To determine the effect of SP-8356 on other signaling pathways which may be involved in cancer progression, STAT3-derived reporter gene expression was examined in IL-6-treated MDA-MB231 cells. Unlike NF-kB, IL-6-dependent STAT3 activation was not inhibited by SP-8356, suggesting that this molecule may downregulate NF-kB signaling with specificity (Fig. 5D). In unstimulated cells, NF-κB predominantly localized to the cytosol, whereas in its active state, the RelA/p65 subunit of NF-κB was translocated to the nucleus. To establish if SP-8356 inhibits the nuclear translocation of p65, we determined protein location by immunostaining with anti-p65 antibodies. Prior to TNF-α treatment, p65 was found mainly in the cytosol but then localized to the nucleus after TNF-α exposure. However, p65 remained in the cytosol of TNF-α-stimulated cells pre-treated with 10 μM SP-8356 (Fig. 5E), and the percentage of cells undergoing p65 nuclear translocation was significantly lower in the presence of SP-8356 and TNF-α compared to cells exposed to TNF-α alone (Fig. 5F).

Because IKK-mediated phosphorylation and degradation of IκB precedes nuclear translocation of NF-κB, SP-8356 may block IκB degradation and indirectly inhibit NF-κB. However, SP-8356 neither changed basal levels of IκB nor blocked TNF-α-stimulated IκB degradation, which indicates SP-8356 may target events following NF-κB’s release from IκB (Fig. 5G). According to previous reports, nuclear translocation of free NF-κB is mediated by a subset of importin molecules that directly bind subunits p50 and p65^22,23^. Immunoprecipitation with cells expressing hemagglutinin (HA)-importin α3 or α5 showed that TNF-α-dependent p65 interactions with importins was significantly inhibited by SP-8356 (Fig. 5H). These findings suggest that SP-8356 negatively regulates TNF-α-induced nuclear translocation of RelA/p65 by interfering with direct interaction between p65 and importin proteins.

### SP-8356 attenuates cell migration-associated gene expression

**Figure 6:**
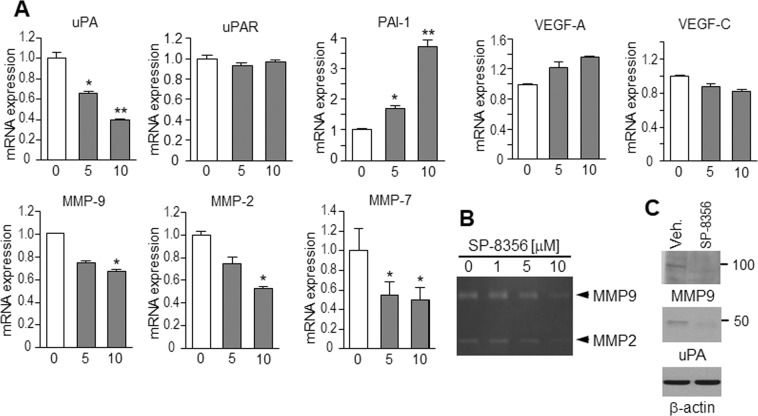
SP-8356 regulates expression of metastasis-related genes. (**A**) The relative mRNA expression levels of *MMP-2*, *MMP-7*, *MMP-9*, *uPA*, *uPAR*, *PAI*, *VEGF-A*, and *VEGF-C* in MDA-MB231 cells treated with varying doses of SP-8356 were evaluated by qRT-PCR. Values are shown as means ± SEM. *p < 0.05, **p < 0.001. (**B**) Effect of SP-8356 on the activity of MMPs. MDA-MB231 cells were treated with SP-8356 for 24 h, and MMP-2 and MMP-9 levels in conditioned culture media were assessed in zymography assays. (**C**) Western blot analysis shows decreased expression of MMP-9 and uPA in MDA-MB21 cells treated with SP-8356. Exposure time for MMP9 and uPA was 1 min. 15 sec. for β-actin.

Metastasis of a primary tumor relies on the tumor cells’ ability to degrade the extracellular matrix (ECM)^24^, so we examined SP-8356′s effect on the expression of genes influencing cell adhesion and invasion targeted by NF-κB. The mRNA levels of *uPA*, *MMP-2*, *MMP-7*, and *MMP-9* in SP-8356-treated MDA-MB231 cells were significantly reduced, whereas *PAI* was elevated compared to control cells (Fig. 6A). Zymography assays revealed that levels of exogenous MMP-2 and MMP-9 were remarkably reduced in the presence of 10 μM SP-8356 (Fig. 6B), and Western blotting showed decreased MMP-9 and urokinase plasminogen activator (uPA) levels in cells treated with 10 μM SP-8356 (Fig. 6C). These results indicate that SP-8356 likely limits the migration and invasion activity of aggressive MDA-MB231 cells by reducing expression of MMPs and uPA and upregulating PAI.

### Pharmacokinetics and concentration-response relationships of SP-8356

Catechol or polyphenol compounds are rapidly metabolized to glucuronides and/or sulfates by phase II reactions, and therefore non-conjugated aglycones are scarcely detected in human plasma^25,26^. SP-8356 with a catechol moiety can be rapidly metabolized by phase II reactions. Thus, we further determined the concentrations of SP-8356 and its metabolites in plasma. After intraperitoneal injection, plasma levels of SP-8356 and its major metabolite SP-8356 monoglucuronide conjugate (Glu-8356) reached to micromolar ranges. The maximal concentration (Cmax) and area under the curve (AUC) of Glu-8356 in plasma were over 11.4 times higher than that of SP-8356 (Fig. S1A). In addition, both SP-8356 and its glucuronide conjugate reduced NF-κB activity to a similar extent (Fig. S1B).

## Discussion

In this study, the verbenone derivative SP-8356 inhibited growth of all breast cancer cell lines tested, which was closely related to cell death or cell cycle arrest. SP-8356 was cytotoxic to these cells by inducing apoptotic molecule activity and halting cell cycle progression at S phase. SP-8356 also showed strong inhibitory effects on wound healing and invasion activity of highly motile TNBC cells. Many signaling pathways that regulate cellular proliferation involve ERK phosphorylation^27^, which declined in cells treated with SP-8356 long-term, although short-term treatment of SP-8356 did not significantly alter basal or serum-stimulated ERK phosphorylation. We also found that SP-8356 slightly reduced serum-dependent SRE activation, a downstream target of ERK whose indirect regulation may contribute SP-8356-mediated growth inhibition of breast cancer cells.

SP-8356 also inhibits transcriptional activity of NF- κB, which induces expression of genes responsible for cell proliferation, survival, and motility. Growth inhibitory effect of SP-8356 was slightly higher than that of JSH-23, another inhibitor of NF-κB transcriptional activity (data not shown). The study on underlying mechanism revealed that SP-8356 negatively regulates NF-κB by blocking its importin-mediated nuclear translocation of p65 rather than inhibiting upstream signaling events of the transcription factor. Thus, regulation of NF-κB and ERK are likely related to SP-8356′s inhibitory action in breast cancer cells, which was generally confirmed in *in vivo* tumor suppression xenograft model. Because NF-κB regulates genes involved in epithelial-mesenchymal transition and metastasis, its inhibition by SP-8356 is extremely relevant to limiting cancer progression. In regard to nuclear translocation of NF-κB, SP-8356 is not likely to act on importin, since it has no effect on STAT3 of which nuclear translocation also requires importin^28^.

In the present study, plasma levels of SP-8356 monoglucuronide conjugate were much higher in comparison to SP-8356. In addition to SP-8356 monoglucuronide, sulfated and methylated meatbolites were also found in plasma levels higher than the parent drug SP-8356 (Data not shown). Like SP-8356 with a catechol moiety, quercetin, a plant flavonol from the flavonoid group of polyphenols, and its water-soluble metabolites, quercetin-3′glucuronide and quercetin-3′-sulfate possess strong anti-proliferative effects^26,29^. Resveratrol, a polyphenolic phytoalexin, and its metabolites, resveratrol-3-O-glucuronide and resveratrol-3-O-sulfate has cell proliferation-inhibiting activities^30^.

Matrix metalloproteases are a family of enzymes capable of degrading various ECM components and facilitating tumor migration^24,31^, and expression of various MMPs is upregulated in many cancers associated with a poor prognosis^32,33^. In addition, uPA binding to its receptor uPAR converts proenzyme plasminogen into active serine protease plasmin^34^, which cleaves ECM proteins and growth factor precursors to their active forms. Ultimately, these growth factors bind their cognate receptors, resulting in cell proliferation and migration^35,36^. Binding of uPA to uPAR also activates JAK-STAT signaling and induces integrin-mediated activation of MAPK and Akt, which leads to cell proliferation, survival, and motility^37–39^. The mRNA expression of these genes (except *uPAR*) was prominently inhibited by SP-8356 in a dose-dependent manner, some of which were confirmed by Western blotting or protease activity assays. Interestingly, plasminogen activator inhibitor (PAI), a serine protease inhibitor, was dramatically enhanced by SP-8356. This molecule normally binds and inhibits the uPA-uPAR complex, induces endocytosis followed by complex degradation^39^, and blocks activity of MMPs^40^. Therefore, positive or negative regulation of genes involved in cell migration and invasion is a possible mechanism of SP-8356′s inhibitory effects against breast cancer cells.

Our recent experiments revealed that SP-8356 binds to CD147 and inhibits dimerization of the protein (unpublished data). CD147 was recently reported to be overexpressed in many human cancers and implicated in tumor progression, especially during proliferation, invasion, and metastasis^41^. As a member of the immunoglobulin superfamily and a type I transmembrane glycoprotein, CD147 is expressed in the plasma membrane as homodimer and responsible for activation of some matrix metalloproteases, which may enhance cell motility^42^. So, targeting to CD147 may contribute to the anti-metastatic activity of SP-8356 of which the mechanism should be verified in the future.

We confirmed the *in vitro* effects of SP-8356 using a xenograft mouse model. Although aggressive TNBC cells initially migrated to the lung and formed large tumors, SP-8356 treatment blocked colonization of the cells and reduced tumor burdens without causing any adverse effects in the mice, further highlighting the efficacy and potential safety of SP-8356 as an anti-cancer agent. Its biological functions related to cell proliferation, survival, and migration may result from its regulation of NF-κB signaling and subsequent expression metastasis-associated genes. Moreover, blocking homophilic interactions of CD147 may be another mechanism for anti-metastatic activity of SP-8356. In summary, SP-8356 is a multi-target agent possessing cancer-specific anti-metastatic activity both *in vitro* and *in vivo*, making it a promising therapy for aggressive breast cancer.

## Materials and Methods

### Reagents, culture media, and antibodies

(1S)-(–)-Verbenone derivatives were synthesized as previously reported^21^. Cell culture media were obtained from WELGENE, Inc. (Daegu, Korea). Human recombinant TNFα was purchased from R&D Systems (Minneapolis, MN, USA), and protease inhibitor cocktail was purchased from Roche (Mannheim, Germany). Antibodies against actin, ERK, NF-κB p65, p-Akt, and p-ERK were purchased from Santa Cruz Biotechnology (Santa Cruz, CA, USA). Antibodies against Akt, PARP, caspase-3 and MMP-9 were obtained from Cell Signaling Technology (Beverly, MA, USA). Antibodies against uPA and CD147 were purchased from Abcam (Cambridge, UK). Primers for gene cloning and materials for expression vector construction were obtained from Cosmogenetech (Seoul, Korea), and DNA sequencing was conducted by the same company. All other reagents were purchased from Sigma-Aldrich (St. Louis, MO, USA) unless otherwise stated.

### Cell culture

MDA-MB231, MDA-MB453, MCF-7, and 4T1 cells were purchased from the American Type Culture Collection (Manassas, VA, USA). All cells were maintained in RPMI-1640 or DMEM supplemented with 10% fetal bovine serum (FBS) and penicillin (100 IU/ml)/streptomycin (100 μg/ml). All cells were cultured at 37 °C in a humidified chamber containing 5% CO_2_.

### Cell growth assay

MDA-MB231, MDA-MB453, MCF-7 (4,000 cells/well) and 4T1 (3,000 cells/well) were seeded into 96-well plates and treated with various concentrations of SP-8356 for indicated times in complete culture media (Fig. 1A). Cell growth was measured using the Cell Counting Kit-8 (CCK-8) from Dojindo Molecular Technologies, Inc. (Rockville, MD, USA) following the manufacturer’s instructions. Cells were incubated with 10 μL CCK-8 solution for 2 h, and the absorbance of each well was then measured at 450 nm using a microplate reader.

### Cell cycle analysis

Cell cycle distribution and apoptosis were determined by FACS analysis using propidium iodide (PI) staining to measure DNA content. Although all cells tested in cell growth were applied for this assay, only MDA-MB231 showed discrete fractions regarding to DNA contents. MDA-MB231 cells were plated at a density of 5 × 10^5^ cells/well in a 6-well-plate. 24 h later, cells were treated with 10 μM SP-8356 every 24 h for three consecutive days. Both adherent and floating cells were harvested, washed with cold PBS, and processed for cell cycle analysis. Briefly, cells were fixed in absolute ethanol and stored in ice for 1 h. The fixed cells were then centrifuged at 500 × *g* for 5 min and washed with PBS. Cells were resuspended with 1 mg/mL PI and 100 μg/mL RNase A and incubated for 30 min at 37 °C. The mixture was then analyzed using the FACSCalibur platform (BD Biosciences; Franklin Lakes, NJ, USA), equipped with CellQuest Pro software.

### Lactate dehydrogenase (LDH) assay

Cell cytotoxicity was quantified by measuring LDH released from plasma membrane-damaged MDA-MB231 cells using a cytotoxicity detection kit according to the manufacturer’s instructions (Takara Bio Company, Shiga, Japan). Briefly, cells were seeded in 96-well plates in RPMI with 10% FBS and incubated for 24 h. Cells were then incubated in 200 μL serum-free RPMI with various concentrations of SP-8356 for 24 h or 48 h (Fig. 1B). Cells treated with DMSO (vehicle) were used as a negative control. Some vehicle-treated cells were lysed with 1% Triton X-100 and used as a positive control. Microtiter plates were centrifuged at 250 × *g* for 10 min, and 100 μL the supernatant was transferred to another 96-well plate followed by the addition of 100 μL reaction mixtures (provided by company). After 30 min incubation at room temperature, the absorbance of each was measured at 490 nm using a microplate reader. The relative activity LDH (%) was calculated as ([A]_sample_ − [A] _negative control)_/([*A*]_high control_ − [*A*]_negative control_)) × 100%.

### Migration and invasion assays

For migration assays, 2 × 10^4^ MDA-MB231 and 4T1 cells in serum-free medium were placed into the upper chamber of transwell insert (8-μm pore size; Corning, Inc.; Corning, NY, USA). For the invasion assays, the upper chamber of an insert was coated with 20 μL 1:6 diluted Matrigel (Invitrogen), which was allowed to solidify, after which 2 × 10^4^ cells in serum-free medium were added and treated with SP-8356. Medium with 10% FBS was added to the lower chamber. Transwell plates were incubated at 37 °C in a humidified chamber containing 5% CO_2_ for 24 h. Cells remaining in the upper membrane were removed with a cotton swab, while cells that migrated or invaded through the membrane were fixed in 4% paraformaldehyde, stained with Hemacolor Rapid staining of blood smear (Merck; Darmstadt, Germany), and quantified microscopically.

### Wound healing assay

5 × 10^5^ MDA-MB231, 4T1, and MDA-MB453 cells were seeded into 6-well plates. Confluent monolayers of cells were scratched with a pipette tip and washed with media to remove floating cells. Cells were then incubated with SP-8356 (1 µM, 2.5 µM, or 5 µM) and visualized after 18 h. The percentage of wound closure was calculated as follows: 100 × (area of original wound − area of remaining wound)/area of original wound.

### Xenograft mouse model

All animal experiments and procedures were performed in accordance with the NIH Guide for the Care and Use of Laboratory Animals and approved by the Ethics Committee and the Institutional Animal Care & Use Committee in Korea University College of Medicine (Approval No. KUIACUC-20150406-2). Five-week-old female NOD/SCID mice were purchased from KOATECH (Pyeongtek, Korea) and housed under specific pathogen-free conditions in an individually ventilated caging system. MDA-MB231 cells (1 × 10^6^ cells) were injected subcutaneously into the right flank of the mice. After 1 day, the mice were randomized into two groups consisting of six mice each. One group was treated with SP-8356 (10 mg/kg) every day by intraperitoneal injection until the end of the experiment, and the other was treated with vehicle (5% DMSO and 10% cremophor in normal saline). Tumor diameter measurement began at >3 mm, and tumor volume was calculated as 0.5 × length × width^2^. When tumor volume reached approximately 2 cm^3^, tumors were surgically resected and weighed.

A lung metastasis model was established by lateral tail vein injection of MDA-MB231 cells (1 × 10^6^ cells) into NOD/SCID mice, which were treated with SP-8356 or vehicle as described above. On day 45, mice were sacrificed, and their lungs were fixed with 4% paraformaldehyde and processed for histological analysis. Sections (5 μm) were mounted on glass slides and subjected to hematoxylin and eosin staining. Representative images for each group were captured, metastatic tumor nodules were counted, and tumor burden was calculated as the percentage of lung area with tumors compared to total lung area.

### Western blot analyses and immunoprecipitation

Cells were lysed in RIPA buffer [50 mM Tris-HCl, pH 7.5, 150 mM NaCl, 1% Triton X-100, 0.5% sodium deoxycholate (w/v), and 0.05% SDS (w/v)] containing protease inhibitors. Protein concentrations of clarified lysates were determined using the Bradford protein assay kit (Bio-Rad; Hercules, CA, USA), and 20 μg cell lysates denatured with SDS sample buffer were separated by SDS-PAGE. Proteins were transferred onto nitrocellulose membranes and probed with appropriate antibodies. Signals were then detected using the ECL assay kit (GE Healthcare; Chicago, IL, USA).

For immunoprecipitation, MDA-MB231 cells transfected with HA-importin genes were lysed with the buffer containing 50 mM Tris-HCl, pH 7.5, 150 mM NaCl, 1% Triton X-100, and protease inhibitors. The clarified cell extracts were incubated with anti-HA agarose for 2 h at 4 °C. After washing, precipitated proteins were applied for SDA-PAGE and Western blotting.

### Luciferase assays

MDA-MB231 cells seeded into 24-well plates were transfected with plasmids containing a *NF-*κ*B-luc*, *STAT3-luc*, or serum responsive element (*SRE*)*-luc* reporter gene. Cells cultured in serum-free medium for 18 h were treated with various assay-specific concentrations of SP-8356 prior to adding serum or TNFα as a stimulant. After 6 h, cells were harvested, and luciferase activity was measured using a standard assay program from BioTek Instruments, Inc. (Winooski, VT).

### Immunocytochemistry

MDA-MB231 cells were fixed with 4% paraformaldehyde and blocked with 3% bovine serum albumin in PBS containing 0.1% Triton X-100 for 30 min. The preparation was then incubated for 2 h at room temperature with RelA/p65 antibody, washed with PBS, and probed with secondary antibody (fluorescein isothiocyanate-conjugated anti-rabbit IgG). Images were captured with a Leica TCS SP5 laser scanning microscope (Wetzlar, Germany).

### Quantitative real time RT-PCR (qRT-PCR)

MDA-MB231 cells were treated with SP-8356 for 24 h in the presence of complete medium. Total RNA was extracted with TRIzol (Invitrogen) according to the manufacturer’s instructions. cDNA was generated using reverse transcriptase from Promega (Madison, WI, USA, and qRT-PCR was performed with iQTM SYBR Green Supermixture and an iCycler PCR thermocycler (Bio-Rad) with gene-specific primer sets designed with Beacon Designer version 2.1 (Biosoft International; Palo Alto, CA, USA) as follows: uPA (5′-ttgctcaccacaacgacatt-3′ and 5′-ggcaggcagatggtctgtat-3′), uPAR (5′-cctctgcaggaccacgat-3′ and 5′-tggtcttctctgagtgggtaca-3′), PAI (5′-actggaaaggcaacatga-3′ and 5′-ctctaggggcttcctgaggt-3′), MMP-2 (5′-cggaaaagattgatgcggta-3′ and 5′-tgctggctgagtagatccag-3′), MMP-7 (5′-gctgacatcatgattggcttt-3′ and 5′-tctcctccgagacctgtcc-3′), MMP-9 (5′-atccggcacctc tatggtc-3′ and 5′-ctgaggggtggacagtgg-3′), VEGF-A (5′-ggagtccaacatcaccat-3′ and 5′-cttgtcttgctctatctttctt-3′), VEGF-C (5′-tcaaggacagaagagactat-3′ and 5′-ctccactcattatcaatacttttc-3′), GAPDH (5′-ctctgctcctcctgttcgac-3′ and 5′-aatccgttgactccgacctt-3′). The mRNA level of each gene was normalized to that of glyceraldehyde 3-phosphate dehydrogenase.

### Zymography

The *in vitro* activities of matrix metalloproteases MMP-2 and MMP-9 from MDA-MB231 cells were analyzed by gelatin zymography using Novex® 10% Zymogram gels (Invitrogen; Carlsbad, CA, USA). After electrophoresis, gels were washed for 1 h at room temperature to remove SDS using 2.5% Triton X-100 (Sigma-Aldrich). The gels were then incubated overnight at 37 °C in zymography reaction buffer containing 50 mM Tris (pH 7.5), 150 mM NaCl, 10 mM CaCl_2_, and 0.02% NaN_3_ and stained with Coomassie Brilliant Blue R-250 (Bio-Rad) followed by destaining with 40% methanol and 10% glacial acetic acid to obtain contrast bands. Images were captured and analyzed by densitometry using ImageJ software (https://imagej.net).

### Statistical analysis

Unpaired Student’s *t*-tests or ANOVA using PRISM5 software (GraphPad; La Jolla, CA, USA) were used. Group means were further analyzed using Bonferroni’s multiple comparison tests. Data were presented as means ± SEM, and all experiments were performed in triplicate unless otherwise indicated.

## Supplementary information

supplementary data

## References

[1] Parkin DM, Fernández LMG (2006). Use of Statistics to Assess the Global Burden of Breast Cancer. The Breast Journal.

[2] Stingl J, Caldas C (2007). Molecular heterogeneity of breast carcinomas and the cancer stem cell hypothesis. Nat Rev Cancer.

[3] Levitzki A, Klein S (2010). Signal transduction therapy of cancer. Molecular aspects of medicine.

[4] Dillman RO (1984). Monoclonal antibodies in the treatment of cancer. Critical reviews in oncology/hematology.

[5] Daub H, Specht K, Ullrich A (2004). Strategies to overcome resistance to targeted protein kinase inhibitors. Nature reviews. Drug discovery.

[6] Xia W (2002). Anti-tumor activity of GW572016: a dual tyrosine kinase inhibitor blocks EGF activation of EGFR/erbB2 and downstream Erk1/2 and AKT pathways. Oncogene.

[7] Maira SM (2008). Identification and characterization of NVP-BEZ235, a new orally available dual phosphatidylinositol 3-kinase/mammalian target of rapamycin inhibitor with potent *in vivo* antitumor activity. Molecular cancer therapeutics.

[8] Biswas DK (2004). NF-κB activation in human breast cancer specimens and its role in cell proliferation and apoptosis. Proceedings of the National Academy of Sciences of the United States of America.

[9] Cui X (2017). NF-κB suppresses apoptosis and promotes bladder cancer cell proliferation by upregulating survivin expression *in vitro* and *in vivo*. Scientific Reports.

[10] Shukla S (2004). Nuclear Factor-κB/p65 (Rel A) Is Constitutively Activated in Human Prostate Adenocarcinoma and Correlates with Disease Progression. Neoplasia (New York, N.Y.).

[11] Nakshatri H, Bhat-Nakshatri P, Martin DA, Goulet RJ, Sledge GW (1997). Constitutive activation of NF-kappaB during progression of breast cancer to hormone-independent growth. Molecular and cellular biology.

[12] Jiang R (2010). High expression levels of IKKα and IKKβ are necessary for the malignant properties of liver cancer. International Journal of Cancer.

[13] Karin M (2006). Nuclear factor-kappaB in cancer development and progression. Nature.

[14] Kuo CF (2011). Anti-inflammatory effects of supercritical carbon dioxide extract and its isolated carnosic acid from Rosmarinus officinalis leaves. J Agric Food Chem.

[15] Ju C (2013). Discovery of novel (1S)-(-)-verbenone derivatives with anti-oxidant and anti-ischemic effects. Bioorg Med Chem Lett.

[16] Dent R (2007). Triple-Negative Breast Cancer: Clinical Features and Patterns of Recurrence. Clinical Cancer Research.

[17] Kassam F (2009). Survival Outcomes for Patients with Metastatic Triple-Negative Breast Cancer: Implications for Clinical Practice and Trial Design. Clinical Breast Cancer.

[18] Paplomata E, O’Regan R (2014). The PI3K/AKT/mTOR pathway in breast cancer: targets, trials and biomarkers. Therapeutic Advances in Medical Oncology.

[19] Santen RJ (2002). The role of mitogen-activated protein (MAP) kinase in breast cancer. The Journal of steroid biochemistry and molecular biology.

[20] Van Antwerp, D. J., Martin, S. J., Verma, I. M. & Green, D. R. Inhibition of TNF-induced apoptosis by NF-kappaB. *Trends in Cell Biology* **8**, 107–111, 10.1016/S0962-8924(97)01215-4. 10.1016/s0962-8924(97)01215-4 9695819

[21] Ju C (2013). Discovery of novel (1S)-(−)-verbenone derivatives with anti-oxidant and anti-ischemic effects. Bioorganic & Medicinal Chemistry Letters.

[22] Fagerlund R, Kinnunen L, Kohler M, Julkunen I, Melen K (2005). NF-{kappa}B is transported into the nucleus by importin {alpha}3 and importin {alpha}4. The Journal of biological chemistry.

[23] Liang P (2013). KPNB1, XPO7 and IPO8 mediate the translocation ofNF-kappaB/p65 into the nucleus. Traffic.

[24] Kumar S, Weaver VM (2009). Mechanics, malignancy, and metastasis: The force journey of a tumor cell. Cancer metastasis reviews.

[25] Day AJ (2001). Human metabolism of dietary flavonoids: identification of plasma metabolites of quercetin. Free Radic Res.

[26] Wu Q (2018). Different antitumor effects of quercetin, quercetin-3′-sulfate and quercetin-3-glucuronide in human breast cancer MCF-7 cells. Food & function.

[27] Chambard J-C, Lefloch R, Pouysségur J, Lenormand P (2007). ERK implication in cell cycle regulation. Biochimica et Biophysica Acta (BBA) - Molecular Cell Research.

[28] Cimica V, Chen HC, Iyer JK, Reich NC (2011). Dynamics of the STAT3 transcription factor: nuclear import dependent on Ran and importin-beta1. PLoS One.

[29] Delgado L (2014). Anti-proliferative effects of quercetin and catechin metabolites. Food & function.

[30] Wang P, Sang S (2018). Metabolism and pharmacokinetics of resveratrol and pterostilbene. BioFactors (Oxford, England).

[31] Kleiner DE, Stetler-Stevenson WG (1999). Matrix metalloproteinases and metastasis. Cancer chemotherapy and pharmacology.

[32] Egeblad, M. & Werb, Z. New functions for the matrix metalloproteinases in cancer progression. *Nat Rev Cancer* **2**, 161–174, http://www.nature.com/nrc/journal/v2/n3/suppinfo/nrc745_S1.html (2002). 10.1038/nrc745 11990853

[33] Kessenbrock K, Plaks V, Werb Z (2010). Matrix Metalloproteinases: Regulators of the Tumor Microenvironment. Cell.

[34] Alfano D (2005). The urokinase plasminogen activator and its receptor: role in cell growth and apoptosis. Thrombosis and haemostasis.

[35] Mazzieri R, Blasi F (2005). The urokinase receptor and the regulation of cell proliferation. Thrombosis and haemostasis.

[36] Gorrasi A (2014). The urokinase receptor takes control of cell migration by recruiting integrins and FPR1 on the cell surface. PLoS One.

[37] Bao Y-N (2014). Urokinase-type plasminogen activator receptor signaling is critical in nasopharyngeal carcinoma cell growth and metastasis. Cell Cycle.

[38] Chen Q, Lin TH, Der CJ, Juliano RL (1996). Integrin-mediated activation of MEK and mitogen-activated protein kinase is independent of Ras [corrected]. The Journal of biological chemistry.

[39] Salvatore U, Enke B, Salvatore S, Massimino DA (2009). The Urokinase Plasminogen Activator System: A Target for Anti-Cancer Therapy. Current Cancer Drug Targets.

[40] Davis GE, Pintar Allen KA, Salazar R, Maxwell SA (2001). Matrix metalloproteinase-1 and -9 activation by plasmin regulates a novel endothelial cell-mediated mechanism of collagen gel contraction and capillary tube regression in three-dimensional collagen matrices. Journal of cell science.

[41] Xiong L, Edwards CK, Zhou L (2014). The Biological Function and Clinical Utilization of CD147 in Human Diseases: A Review of the Current Scientific Literature. International Journal of Molecular Sciences.

[42] Huang W (2013). Modulation of CD147-induced matrix metalloproteinase activity: role of CD147 N-glycosylation. The Biochemical journal.

