# Supplementary material for: SP-8356, a (1S)-(–)-verbenone derivative, exerts in vitro and in vivo anti-breast cancer effects by inhibiting NF-κB signaling
Source: Sci Rep. 2019 Apr 29;9:6595. doi: 10.1038/s41598-019-41224-y (PMC6488667; doi:10.1038/s41598-019-41224-y)
Supplement: Supplementary file 1 — supplementary data [file 41598_2019_41224_MOESM1_ESM.pdf]

SP-8356, a (1S)-(-)-verbenone derivative, exerts *in vitro* and *in vivo* anti-breast cancer effects by inhibiting NF-κB signaling

Sunam Mander[1], Dong Hwi Kim[1], Huong Thi Nguyen[1], Hyo Jeong Yong[1], Kisoo Pahk[2,3,4], Jae Young Seong[1], Eun-Yeong Kim[5], Kiho Lee[5,6], Won-Ki Kim[1,2,3*], and Jong-Ik Hwang[1*]

[1, 2]Departments of Biomedical Sciences[1] and Neuroscience[2], College of Medicine, Korea University, 73 Inchon-ro, Seongbuk-gu, Seoul 136-705, Republic of Korea

[3]Institute of Inflammation Control, Korea University, 73 Inchon-ro, Seongbuk-gu, Seoul 02841, Republic of Korea

[4]Department of Nuclear Medicine, Korea University, Anam Hospital, 73 Inchon-ro, Seongbuk-gu, Seoul 02841, Republic of Korea

[5]College of Pharmacy, Korea University, Sejong 30019, Republic of Korea

[6]Biomedical Research Center, Korea University Guro Hospital, Seoul 08308, Republic of Korea

[*]Corresponding authors: Jong-Ik Hwang: Phone: 82-2-2286-1093; E-mail: hjibio@korea.ac.kr and Won-Ki Kim: Phone: 82-2-2286-1094; E-mail: wonki@korea.ac.kr.

Supplementary methods

Pharmacokinetics of SP-8356 and SP-8356-glucuronide conjugate (Glu-8356) in mice following a single intraperitoneal dose of SP-8356

Female ICR mice (8 weeks old, 25~30 g) were acclimated to the testing facility in a temperature and humidity-controlled condition for approximately a week prior to the study. The dosing solution was prepared at 5 mg/mL in DMSO/10% Cremophor EL in saline (5/95 vol%). The

dosing solution was dosed by intraperitoneal injection at a dose of 50 mg/kg and a dose volume of 10 ml/kg. About 40 μl of blood samples were collected into Microvette®100LH capillary tubes (Sarstedt AG & Co., Nümbrecht, Germany) at selected times through the saphenous vein over 24 h post-dosing. Blood samples were centrifuged at 6000 x g for 5 min to separate plasma and stored in a freezer until analyzed. Protein precipitation was conducted on 15 μl aliquot of the plasma samples with 3 volumes of acetonitrile containing a SP-8356 derivative LMT-311 as analytical internal standard. After centrifugation at 3000 x g for 30 min at 4°C, a 50 μl aliquot of supernatant was transferred to a 96-well plate and mixed with 50 μl of deionized water. The resulting solutions were analyzed by Agilent 6460 QQQ LC-MS/MS system (Agilent Technologies, Inc., Santa Clara, CA) in a negative MRM mode. Pharmacokinetic parameters were estimated by noncompartmental analysis of the plasma concentration-time curves using PKSolver.

Supplementary figure legends

Fig. S1. A) Mean plasma concentration-time curves of SP-8356 and its metabolite SP-8356-glucuronide conjugate (Glu-8356) following a single i.p. dose (50 mg/Kg) of SP-8356 in mice. Data are mean±SD (n=3). PK parameters were calculated by non-compartment analysis using PKSolver. B) Concentration-response relationships between SP-8356 and Glu-8356 for NF-κB activity suppression. MDA-MB231 cells were transiently transfected with a NF-κB-luciferase reporter gene. After 24 h of serum starvation, cells were pre-treated for 30 min with SP-8356 or SP-8356 glucuronide conjugate (Glu-8356) and stimulated with 10 ng/ml TNF-α for 6 h. Cell lysates were then assayed for luciferase activity. Values are shown as means ± SEM. * $p < 0.05$, ** $p < 0.001$: compared to TNF-α stimulation alone (hatched bar); ## $p < 0.001$: compared to control (open bar)

Fig. S2. Quantitative analysis for western blot data Fig.1D, Fig. 4E, Fig. 5H, and Fig. 6C. *$p < 0.05$, **$p < 0.001$ (compared to control)

A

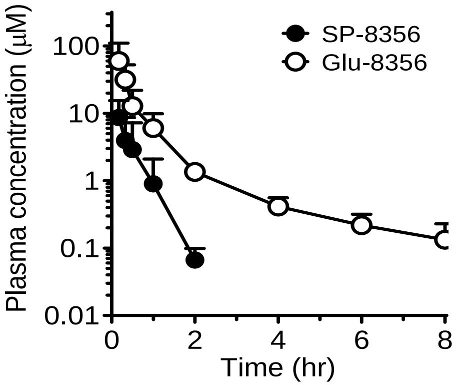

Parameters	SP-8356	Glu-8356
$t_{1/2}$ (h)	0.6±0.4	2.0±0.5
t_{max} (h)	0.17±0.0	0.22±0.09
C_{max} (μM)	8.65±6.85	61.97±47.04
AUC_{last} (μM·h)	3.68±4.11	27.39±14.91
AUC_{inf} (μM·h)	3.75±4.06	27.79±14.71
MRT (h)	0.6±0.2	1.1±0.6

B

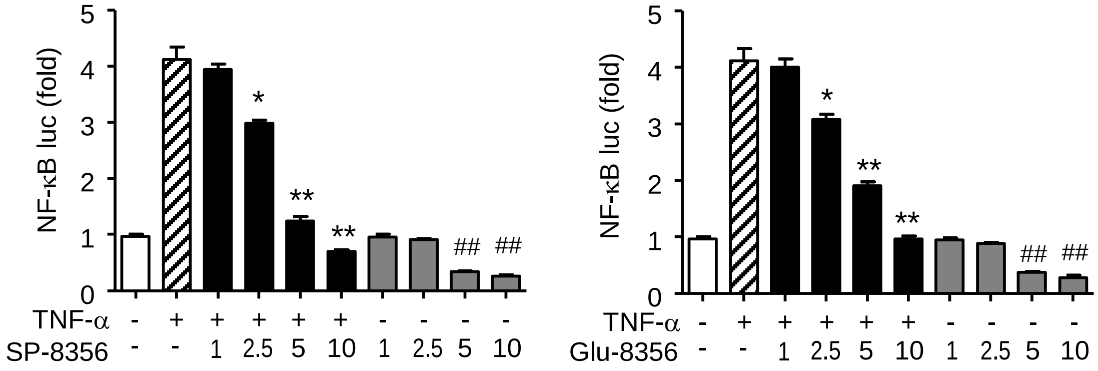

Fig. S1 Plasma concentration time of of SP-8356 and its metabolite, and their inhibitory effect on NF-κB activation

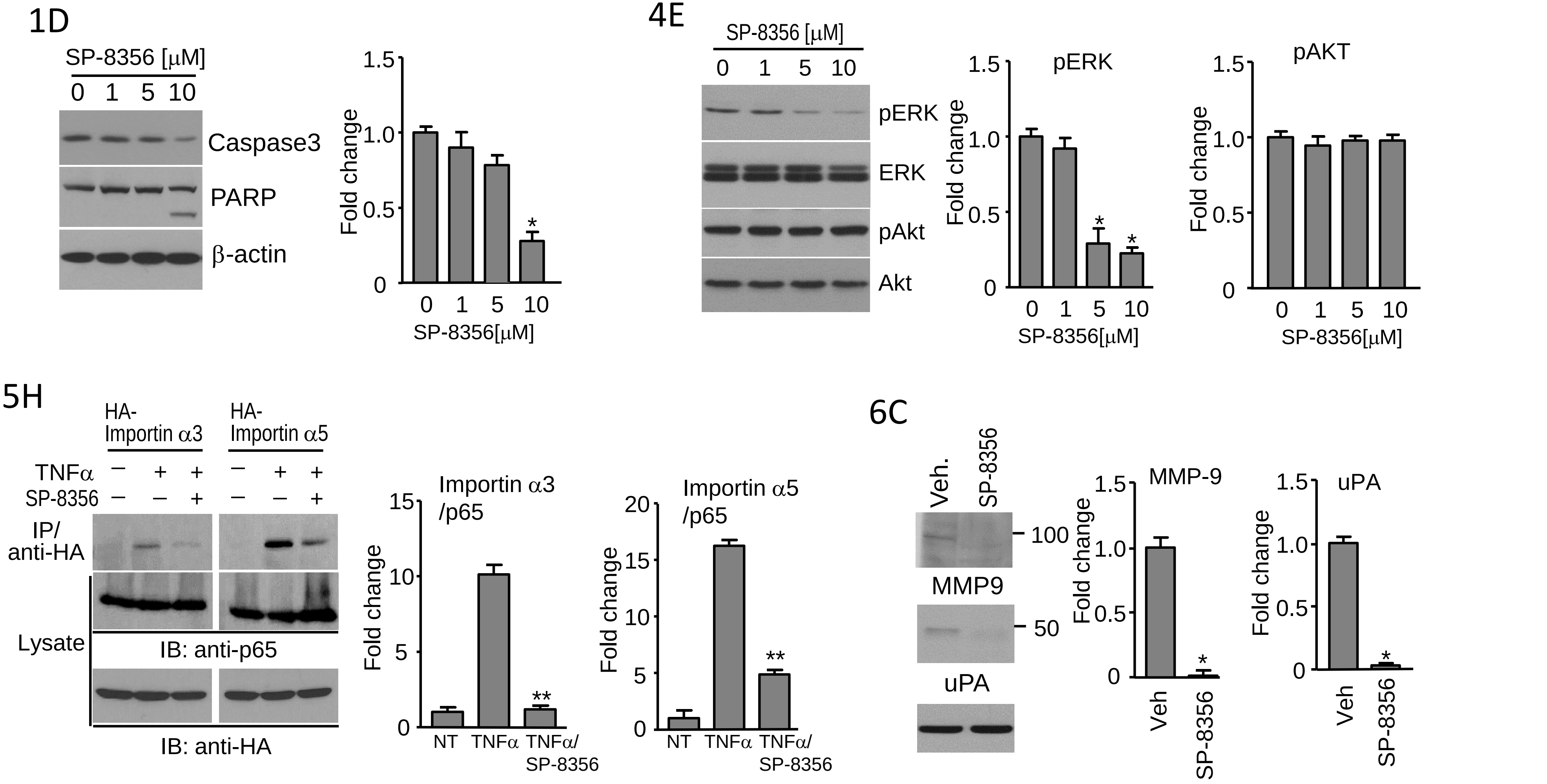

Fig. S2 Quantitative analysis for western blot data Fig.1D, Fig. 4E, Fig. 5H, and Fig. 6C
